# Rapid Strategy for Screening by Pyrosequencing of Influenza Virus Reassortants - Candidates for Live Attenuated Vaccines

**DOI:** 10.1371/journal.pone.0092580

**Published:** 2014-03-19

**Authors:** Svetlana V. Shcherbik, Nicholas C. Pearce, Marnie L. Levine, Alexander I. Klimov, Julie M. Villanueva, Tatiana L. Bousse

**Affiliations:** 1 Virology, Surveillance and Diagnosis Branch, Influenza Division, National Center for Immunization and Respiratory Diseases, Centers for Disease Control and Prevention, Atlanta, Georgia, United States of America; 2 Battelle, Atlanta, Georgia, United States of America; Johns Hopkins University - Bloomberg School of Public Health, United States of America

## Abstract

**Background:**

Live attenuated influenza vaccine viruses (LAIVs) can be generated by classical reassortment of gene segments between a cold adapted, temperature sensitive and attenuated Master Donor Virus (MDV) and a seasonal wild-type (*wt*) virus. The vaccine candidates contain hemagglutinin (HA) and neuraminidase (NA) genes derived from the circulating *wt* viruses and the remaining six genes derived from the MDV strains. Rapid, efficient selection of the viruses with 6∶2 genome compositions from the large number of genetically different viruses generated during reassortment is essential for the biannual production schedule of vaccine viruses.

**Methodology/Principal Findings:**

This manuscript describes a new approach for the genotypic analysis of LAIV reassortant virus clones based on pyrosequencing. LAIV candidate viruses were created by classical reassortment of seasonal influenza A (H3N2) (A/Victoria/361/2011, A/Ohio/02/2012, A/Texas/50/2012) or influenza A (H7N9) (A/Anhui/1/2013) *wt* viruses with the MDV A/Leningrad/134/17/57(H2N2). Using strain-specific pyrosequencing assays, mixed gene variations were detected in the allantoic progenies during the cloning procedure. The pyrosequencing analysis also allowed for estimation of the relative abundance of segment variants in mixed populations. This semi-quantitative approach was used for selecting specific clones for the subsequent cloning procedures.

**Conclusions/Significance:**

The present study demonstrates that pyrosequencing analysis is a useful technique for rapid and reliable genotyping of reassortants and intermediate clones during the preparation of LAIV candidates, and can expedite the selection of vaccine virus candidates.

## Introduction

The influenza virus is a globally important respiratory pathogen which causes significant morbidity and mortality in humans and animals. Influenza vaccination is the most effective method for preventing influenza virus infection and its potentially severe complications [1], [2]. Accumulation of mutations in genes encoding for the viral surface proteins leading to antigenic drift require that the WHO recommendations for influenza vaccine viruses be updated on a biannual basis to provide protection against contemporary seasonal influenza virus strains. There are two major types of influenza vaccines licensed for human use – inactivated influenza vaccine (IIV), which is injected intramuscularly or intradermally, and live attenuated influenza vaccine (LAIV), which is administered intranasally. LAIVs have previously been shown to be as effective as IIVs [3], [4]. In some studies, LAIVs appeared to be more effective in preventing influenza infection than trivalent IIV [1], [5]–[8].

The viruses in LAIVs are 6∶2 reassortants in which six internal genes (PB2, PB1, PA, NP, M, and NS) are derived from a cold-adapted (*ca*) and temperature sensitive (*ts*) Master Donor Virus (MDV) and the surface antigen genes, HA and NA, are derived from the circulating wild-type viruses recommended by the World Health Organization (WHO) for seasonal vaccine production. The six genes from the MDV strains provide the attenuated phenotype, and the HA and NA genes from the wild-type viruses confer the protective immunity against contemporary influenza strains. LAIVs based on Russian MDV strains are prepared by classical reassortment in embryonated eggs. Influenza A vaccines are based on A/Leningrad/134/57 (H2N2) virus which underwent 17 passages in eggs at 25–26°C, resulting in the *ca* A/Leningrad/134/17/57 MDV [9], [10]. The live attenuated vaccines based on this virus and the influenza B donor virus, B/USSR/60/69 [11], generated by the Institute of Experimental Medicine (IEM, St. Petersburg, Russia) have been used in Russia in adults since 1980 and in all age groups since 1987 [12], [13]. A high level of safety has been reported in both pediatric and adult populations receiving the Russian LAIV [8]. Recently the Russian LAIVs were licensed to the WHO for the subsequent transfer of the technology to developing country manufacturers who could then provide influenza vaccines to the public royalty-free [13]. The increased international demand of Russian LAIV reassortant viruses prompted the WHO and IEM to establish a back-up facility at the Centers of Disease Control and Prevention (CDC), Influenza Division to optimize and prepare LAIV reassortants for international use.

LAIV produced by co-infection results in a pool of reassortant viruses with a random combination of the eight RNA genomes from the two parental viruses which then are subjected to passages under selective pressure (in the presence of serum to MDV and at low temperature). However, during preparation of LAIV candidates, even under selective pressure, variability in genes donated by the *ca* donor of both influenza A and B was reported [14]–[18]. Reassortant populations of *ca* donors other than 6∶2 genome combinations - with 7∶1(HA from wt and the rest from MDV) and 5∶3 composition (HA, NA and M or NS from *wt* virus and the rest of 5 genes from MDV) - were found to be prevalent [18], [19]. As a result, a sensitive genotyping method is required for the rapid identification and isolation of the reassortant clone containing the desired gene composition to enable vaccines to be manufactured in a timely manner.

Several methods have been described and used for the screening and genotyping of reassortant influenza viruses, such as analysis of restriction fragment length polymorphism (RFLP) of viral genes produced by reverse-transcription polymerase chain reaction (RT-PCR) [20]–[24], and multiplex RT-PCR techniques [25]–[28]. However, none of these techniques provide genetic sequencing data. Sometimes, in the case of high sequence homology between the genes of viruses used in reassortment the genotyping by RT-PCR-RFLP cannot be performed because the lack of suitable restriction sites in the genome [29]. Pyrosequencing has previously been used for genotyping herpes simplex, hepatitis C viruses [30], [31] It has also been used for diagnostic applications, identification and subtyping of emerging influenza A viruses and influenza A reassortants including LAIV candidate viruses [32], for monitoring of drug resistance in seasonal influenza A viruses [33]–[37] and for detection and differentiation of currently circulating human B-lineage viruses [38]. Pyrosequencing differs from other sequencing technologies in its ability to generate quantitative data from the nucleotide incorporation which allows for an accurate analysis and detection of minor variants in a population [39]–[41]. In addition, pyrosequencing is a rapid technique which allows screening of a large number of samples.

In the present study we applied the pyrosequencing technology for the screening of the reassortant clones between seasonal influenza A (H3N2) or influenza A (H7N9) *wt* viruses and MDV of influenza type A viruses created during LAIV generation. The proposed approach can be used as a rapid and reliable genotyping technique for the preparation of LAIV candidates and expedite the generation of vaccine.

## Materials and Methods

### Viruses

A/Leningrad/134/17/57 (H2N2) was provided by IEM (St. Petersburg, Russia). *Wt* influenza viruses, A/Victoria/361/2011 (H3N2), A/Ohio/02/2012 (H3N2), A/Texas/50/2012 (H3N2) and A/Anhui/1/2013 (H7N9), which were adapted to grow in embryonated chicken eggs, were obtained from the Virus Reference Laboratory of Influenza Division of CDC (Atlanta, GA, USA). All viruses were propagated in 10-day-old specific pathogen free (SPF) eggs (Charles River Laboratories Inc., Wilmington, MA). All experiments on reassortment between influenza A (H7N9) virus and MDV were performed in an approved biosafety level 3 (BSL-3) containment laboratory.

### Hemagglutination Inhibition (HI) Assay

The HI assay was used to determine the origin of hemagglutinin (HA) genes of reassortant virus clones. HI assays were performed in 96-well V-microtiter plates using 0.5% turkey red blood cells and antiserum against MDV or *wt* influenza viruses [42].

### Reassortment of MDV and *wt* Influenza Viruses

**Figure 1:**
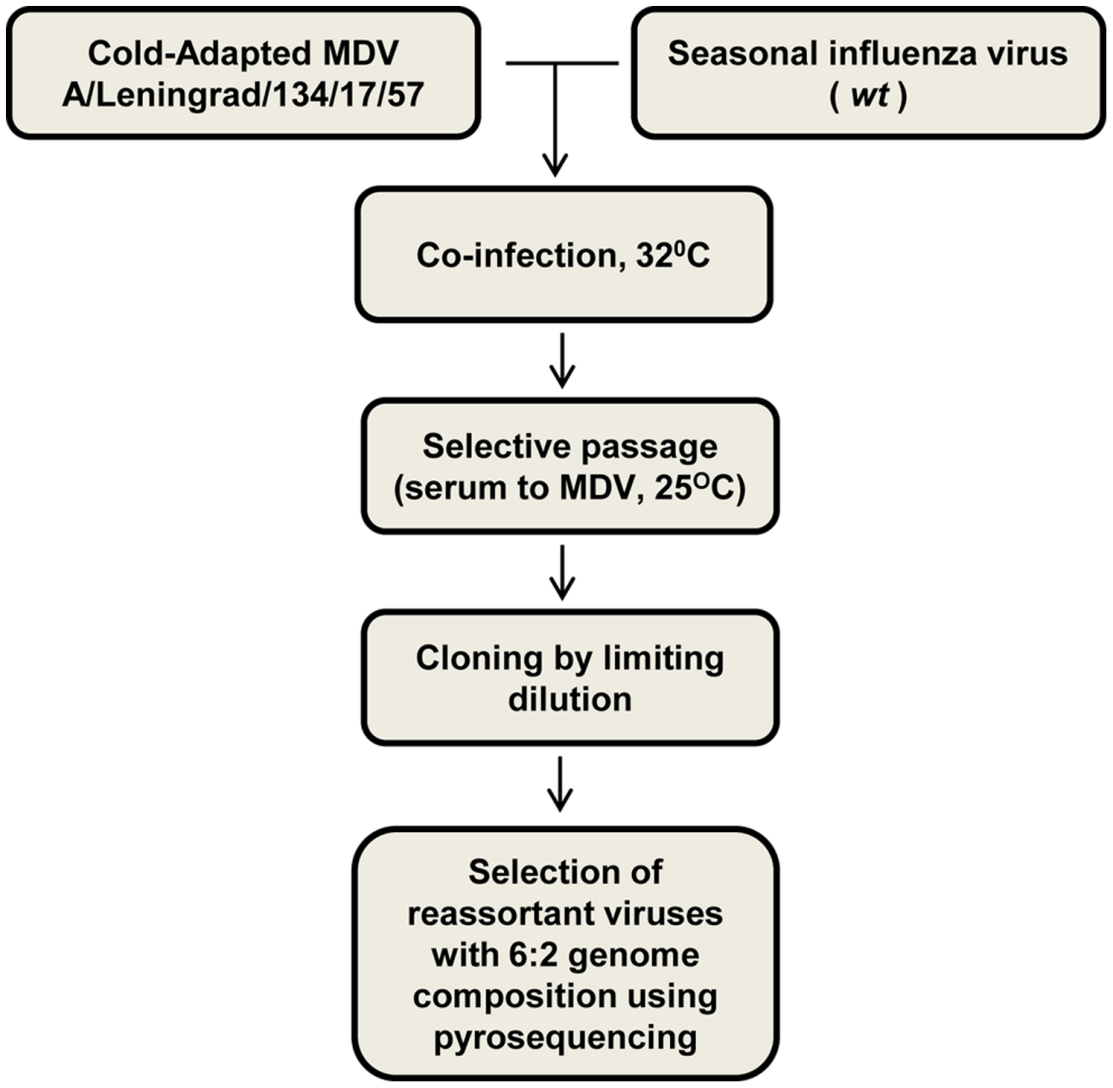
Diagram of LAIV production by classical reassortment and cloning.

Reassortant influenza viruses that possess the internal genes of MDV and the surface antigen genes of *wt* viruses used were prepared according to the method developed by IEM, St. Petersburg, Russia [43]–[45]. Briefly, as outlined in Figure 1, donor and *wt* viruses were inoculated into 10-day-old SPF eggs and incubated at 32°C for 2 days. HA-positive allantoic fluids (AFs) were combined and diluted 1∶10 using antiserum prepared against the MDV in ferrets. The virus-serum mixtures were incubated overnight at 4°C and then passaged once in SPF eggs at 25°C for 6 days. If virus HA titer was not detectable, a blind passage at 32°C was performed. HA-positive AFs were analyzed by HI assay for antigenic specificity with antiserum to MDV and *wt* influenza virus. AFs which exhibited antigenic specificity of *wt* virus were combined and a cloning procedure was carried out using 10 day-old SPF eggs in the presence of antiserum at 25°C as described in [44]. Each clone was analyzed for genome composition using the pyrosequencing assay.

### Design of Primers

Full length sequences of genes of cloned MDV A/Leningrad/134/17/57 virus were provided by IEM (St. Petersburg, Russia). The sequences of the *wt* viruses were obtained from the Influenza CDC Sequence Database. The targets with signature nucleotides which were strain-specific for each gene were selected using BioEdit Sequence Alignment Editor. To amplify and analyze target sequences, RT-PCR and sequencing primers were designed using the Pyrosequencing Assay (PSQ) Design software, version 1.0.6 (Qiagen). The RT-PCR primers used for genotyping influenza A H3N2 and H7N9 reassortants are shown in Table 1 and Table 2, respectively; sequencing primers are shown in Table 3 and Table 4. The primers were synthesized at the CDC Biotechnology Core facility.

**Table 1:** Oligonucleotide primers used for RT-PCR amplification of MDV and H3N2 viruses.

Oligo ID	Gene	Nucleotide region	Direction	Sequence 5′ ->3′
**NA-1246F**	NA	1246–1268	Forward	AGCTGCATCAATMGGTGCTTTTA
**NA-1334R**	NA	1334–1353	Reverse	Biotin-ACCTGARGTGCCACAAAACA
**NS-354F**	NS	354–372	Forward	AATGGACCAGGCAATCATG
**NS-458R**	NS	436–458	Reverse	Biotin-TCTTCGGTGAAAGCCCTTAGTAA
**M-787F**	M	787–805	Forward	TGGGATCTTGCACTTGATA
**M-873R**	M	854–873	Reverse	Biotin-TCTTTTMAGRCCGTGTTT
**PB1-38F**	PB1	38–56	Forward	Biotin-CAGCGCAAAATGCCATAAG
**PB1-219R**	PB1	196–219	Reverse	TAGTGGTCCATCAATTGGGTTRAG
**PB2-120F**	PB2	120–143	Forward	AAAGAACCCGTCACTTAGGATGAA
**PB2-308R**	PB2	285–308	Reverse	Biotin-CCATTYCTATTCCACCATGTYACA
**PA-66F**	PA	66–88	Forward	AGAGTATGGRGAGGATCYGAAAA
**PA-328R**	PA	308–328	Reverse	Biotin-ACAAATCYGGYAGAAACTTCG
**NP-36F**	NP	36–56	Forward	Biotin-GATGGAAACTGATGGGGAWCG
**NP-140R**	NP	118–140	Reverse	AGTTCRGTGCACATTTGGATGTA

**Table 2:** Oligonucleotide primers used for RT-PCR amplification of MDV and H7N9 viruses.

Oligo ID	Gene	Nucleotide region	Direction	Sequence 5′ ->3′
**NA-Len-UTR-F**	NA	1–19 (5′ UTR)	Forward	AGCAAAAGCAGGAGTGAAA
**NA-N9-UTR-F**	NA	1–18 (5′ UTR)	Forward	AGCAAAAGCAGGGTCAAG
**NA-H7N9-374R**	NA	353–374	Reverse	Biotin-TCGCATGAMACATAAGRTTCTC
**M-H7N9-787F**	M	787–805	Forward	TGGGATMTTGCACTTGATA
**M-H7N9-873R**	M	854–873	Reverse	Biotin- CCTCTTTTCARACCGTRTTT
**NS-H7N9-43F**	NS	43–61	Forward	Biotin-CTTTGGCATGTCCGCAAAC
**NS-H7N9-124R**	NS	105–124	Reverse	ACTTCTGATCTCGGCGAAGC
**NP-H7N9-1269F**	NP	1269–1286	Forward	AACCATYATGGCAGCATT
**NP-H7N9-1352R**	NP	1333–1352	Reverse	Biotin-GCACYTTCCATCATYCTTAT
**PA-H7N9-526F**	PA	526–548	Forward	Biotin-TTCACCATAAGRCARGAAATGGC
**PA-H7N9-600R**	PA	581–600	Reverse	TGTTTCTTCGCCTCTTTCRG
**PB1-H7N9-211F**	PB1	211–233	Forward	Biotin-GGACCAYTACCTGAGGACAAYGA
**PB1-H7N9-293R**	PB1	271–293	Reverse	GATTCTTCAAGGAAAGCCATTGC
**PB2-H7N9-27F**	PB2	27–49	Forward	Biotin-TYTGATGTCRCAGTCTCGCACTC
**PB2-H7N9-160R**	PB2	140–160	Reverse	TCATTGCCATCATCCAYTTCA

**Table 3:** Sequencing Primers for genotyping by pyrosequencing of MDV and H3N2 viruses.

Oligo ID	Virus	Gene	Nucleotides	Sequence 5′ ->3′
**NA-Pseq-1272F** *	H3N2	NA	1272–1288	GGAGTTGATWAGGGGAA
**NS-Pseq-395F**	H3N2	NS	395–414	CGAATTTCARTGTGATTTTT
**M-Pseq-820F**	H3N2	M	820–837	TCGTCTTTTTTTCAAATG
**PB1-Pseq-156R**	H3N2	PB1	156–170	GTYGTCCACTTCCCC
**PB2-Pseq-151F**	H3N2	PB2	151–167	ATGGCAATGAAATATCC
**PA-Pseq-106F**	H3N2	PA	106–121	GCAGCAATATGCACTC
**NP-Pseq-79R**	H3N2	NP	79–93	CTTCCCGRCGGATGC

*F and R in the primer name indicate forward and reverse direction, respectively.

**Table 4:** Sequencing Primers for genotyping by pyrosequencing of MDV and H7N9 viruses.

Oligo ID	Virus	Gene	Nucleotides	Sequence 5′ ->3′
**NA-H7N9-1F** *	H7N9	NA	1–20	ATGAATCCAAATCARAAGAT
**M-H7N9-820F**	H7N9	M	820–839	TCGTCTTTTYTTCAAATGCA
**NS-H7N9-105R**	H7N9	NS	105–119	TGATCTCGGCGAAGC
**NP-H7N9-1272F**	H7N9	NP	1272–1286	CATYATGGCAGCATT
**PA-H7N9-562R**	H7N9	PA	562–576	ACGAAAGGAATCCCA
**PB1-H7N9-267R**	H7N9	PB1	267–281	AAAGCCATTGCTTCC
**PB2-H7N9-134R**	H7N9	PB2	134–151	TCATCCAYTTCATCCTAA

*F and R in the primer name indicate forward and reverse direction, respectively.

### RNA Extraction and RT-PCR

Viral RNAs were extracted from 200 μl of allantoic fluids of infected eggs using the MagNA Pure Total Nucleic Acid Kit (Roche) and the MagNa Pure LC 2.0 Robot (Roche). RNA was eluted in a final volume of 50 μl. SuperScript^TM^III One-Step HiFi System (Invitrogen) was used to produce RT-PCR products of all genes except for the HA (previously determined by the HI assay) for pyrosequencing. Primers were used at a final concentration of 0.4 μM. The RT-PCR conditions were 50°C for 30 minutes, denaturation at 94°C for 2 minutes, followed by 45 cycles of 94°C for 15 seconds, annealing 55°C for 30 seconds, 68°C for 1 minute. The RT-PCR products were examined on a 96-well, 2% agarose E-gel (Invitrogen) to confirm amplification of an appropriately sized DNA band. A negative control (water) was used to determine the level of background associated with the primers. Nucleotide dispensations for the pyrosequencing assay were customized to improve the detection of strain-specific nucleotide differences [39]. The modified dispensation CCATTGCAAGCCAATGCCAATGCATG was used for all genes of influenza A (H3N2) influenza viruses. The dispensation orders for influenza A (H7N9) genes were as follows: NA, TACTATGCACTGCAGTC; NS, CGATCTAGATG; M, ATATCGCTCGTTA; NP, CTACTAGATGACATGA; PA, GTAGGACTCTGATAGC; PB1, AGTACGACAGTCTGTG PB2, GTGACAGATCTCTC.

### Pyrosequencing

Pyrosequencing was performed on a PyroMark Q96 ID instrument using the manufacturer’s protocol (Qiagen). In brief, 10 μl of biotinylated PCR product was immobilized onto Streptavidin Sepharose High Performance beads (Amersham Biosciences) in binding buffer (10 mM Tris-HCl, pH 7.6, 2 M NaCl, 1 mM EDTA, and 0.1% Tween 20) and vigorously shaken for 10 min at room temperature. Single-stranded DNA template was obtained by using the PyroMark vacuum prep workstation (Qiagen). The immobilized PCR product was initially washed in 70% ethanol and then in 0.2 M NaOH. A final wash in 10 mM Tris-acetate, pH 7.6, was carried out before annealing the now single-stranded DNA to the sequencing primer. The sequencing primer (440 nM) in annealing buffer (20 mM Tris-acetate, pH 7.6 and 2 mM MgAc_2_) was incubated at 89°C for 2 min and then allowed to cool slowly to room temperature. Pyrosequencing reactions were performed with PyroMark Gold (Qiagen) using an automated PSQ PyroMark ID instrument (Qiagen). Only the Sequence Analysis (SQA) mode was utilized. Sequence results were obtained in the form of pyrograms and analyzed using visual interpretation and the PyroMark Q96 software (Qiagen). Raw pyrogram data obtained during preparation of influenza virus reassortants - candidates for live attenuated vaccines. Could be found at figshare http://dx.doi.org/10.6084/m9.figshare.907510.

## Results

### Development and Evaluation of Pyrosequencing Assays

**Figure 2:**
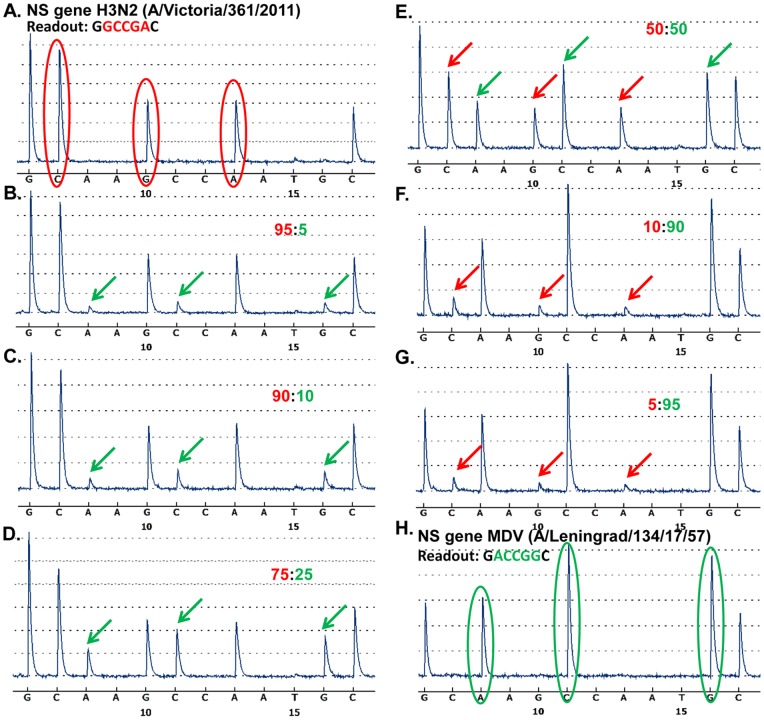
Detection limit of minor viral RNA in mixed populations. The defined mixtures of *wt* A/Victoria/361/2011(H3N2) and MDV A/Leningrad/134/17/57(H2N2). RNAs were analyzed by pyrosequencing of NS gene. The signature nucleotides of *wt* are shown in red, MDV – in green. The analysis was carried out with (A) 100% *wt*, mixtures of *wt*:MDV - (B) 95∶5, (C) 90∶10, (D) 75∶25, (E) 50∶50, (F) 10∶90, (G) 5∶95) and (H) 100% of MDV RNA.

The RT-PCR fragments, amplified with pyrosequencing primers designed for each gene of the MDV-H3N2 and the MDV-H7N9 pairs (Tables 1 and 2), contained the targets with strain-specific signature nucleotides. The target sequences for MDV-H3N2 and MDV-H7N9 genotyping are shown in Table 5 and Table 6, respectively. The unique strain-specific nucleotides are highlighted in red. Primer design and gene-specific pyrosequencing assays were validated using RNAs isolated from MDVs and *wt* viruses. RT-PCR products analyzed on an agarose gel, showed a clear, single band without non-specific products or primer-dimers. The pyrosequencing runs of these RT-PCR fragments showed that all the developed assays were specific for *wt* and *ca* viruses. Strain-specific nucleotide peaks were detected with little to no background noise, allowing discrimination between the parental strains of viruses used in reassortment. The examples of obtained pyrograms for MDVs and *wt* viruses are shown on Fig. 2 A and H.

**Table 5:** Target sequences for genotyping by pyrosequencing of MDV and H3N2 viruses.

Virus	Gene	Nucleotides	Sequence 5′ ->3′
**MDV**	NA	1289–1304	G**GCC**A**C**AGGA**G**ACT**AG** *
**A/Victoria/361/2011**	NA	1289–1304	G**AAA**A**G**AGGA**A**ACT**GA**
**A/Ohio/02/2012**			G**AAA**A**G**AGGA**A**AC**AGA**
**A/Texas/50/2012**			G**AAA**A**G**AGGA**A**AC**TGA**
**MDV**	M	838–849	C**AAT**TATCG**CT**T
**H3N2**	M	838–849	C**GTC**TATCG**AC**T
**MDV**	NS	415–422	G**A**CCG**G**CT
**H3N2**	NS	415–422	G**G**CCG**A**CT
**MDV-**	PB1	140–155	**T**T**T**TCTGAATATTGAT**
**H3N2**	PB1	140–155	**C**T**C**TCTGAATATTG**G**T**
**MDV**	PB2	168–174	**G**AT**T**AC**A**
**H3N2**	PB2	168–174	**A**AT**C**AC**T**
**MDV**	PA	122–132	A**T**TTGGA**A**GT**A**
**H3N2**	PA	122–132	A**C**TTGGA**G**GTG
**MDV**	NP	71–78	**T**CT**G**AT**T**T**
**A/Victoria/361/2011**	NP	71–78	**C**CT**A**AT**C**T**
**A/Ohio/02/2012**			**C**CT**T**AT**C**T**
**A/Texas/50/2012**			**C**CT**A**AT**C**T**

*Signature nucleotides are shown in bold.

**The sequence is of the complementary strand.

**Table 6:** Target sequences for genotyping by pyrosequencing of MDV and H7N9 viruses.

Virus	Gene	Nucleotides	Sequence 5′ ->3′
**MDV**	NA	21–35	**AA**TAA**C**AATT**GG**C**T**C*
**A/Anhui/1/2013**	NA	21–35	**TC** TA**TG**CACTTC**AG**C
**MDV**	M	840–855	**A** TTATCG**CTTC**TTTAA
**A/Anhui/1/2013**	M	840–855	**TTT** ATCG**TCG**TTTTAA
**MDV**	NS	92–104	CG**A**TC**AA**GGAATG**
**A/Anhui/1/2013**	NS	92–104	C**GG**TC**T**AGAAATG**
**MDV**	NP	1287–1301	**C** AC**T**GGGAAT**G**C**A**GA
**A/Anhui/1/2013**	NP	1287–1301	**T** AC**A**GGAAAT**A**C**T**GA
**MDV**	PA	546–561	**G**AG**G**CCT**C**TGCT**A**GCC**
**A/Anhui/1/2013**	PA	546–561	**T**AG**A**CCCCT**G**CT**G**GCC**
**MDV**	PB1	250–266	A**GG**AC**G**CA**G**TC**T**GTTTG**
**A/Anhui/1/2013**	PB1	250–266	AA**T**AC**A**CAATCCGTTTG**
**MDV**	PB2	117–133	G**T**G**AC**GGGTTCTTTTCC**
**A/Anhui/1/2013**	PB2	117–133	GGG**CA**GG**A**TTCTT**C**TCC**

*Signature nucleotides are shown in bold.

**The sequence is of the complementary strand.

To analyze the sensitivity of developed pyrosequencing assays, RNA from MDV A/Leningrad/134/17/57 and *wt* H3N2 virus A/Victoria/361/2011 were mixed in defined proportions based on known virus infectivity (EID_50_/ml) from 100% of *wt* to 100% of MDV in 95∶5, 90∶10, 75∶25, 50∶50, 25∶75, 10∶90, 5∶95 (*wt*:MDV) mixtures. The pyrosequencing assay for the NS gene was used to evaluate the limit of detection of minor viral RNA in mixtures. The peak heights in the pyrograms are proportional to the number of each nucleotide incorporated and also to the percentage of each RNA species present in the mixture. The pyrosequencing analysis indicated that the detection level of minor RNA within the mixed viral RNA population under these assay conditions was at least 5% (Fig. 2).

### Screening of the Reassortant Viruses by Pyrosequencing

To produce the LAIV reassortants, embryonated eggs were co-infected with *wt* virus and MDV. The reassortant viruses containing the desired gene combination 6∶2 were further selected and cloned as outlined in Figure 1. The pyrosequencing assays were utilized for the screening of reassortant clones produced during LAIV preparation. Reassortant progeny which were inhibited by serum to *wt* virus in HI assay (see Materials and Methods), i.e. containing the *wt* HA gene, were subjected to genotyping by pyrosequencing. The 96 well format of pyrosequencing allowed us to analyze the origin of three genes in 30 clones in one run. At first, the reassortants were analyzed for the origin of NA, M and NS genes since 7∶1 (HA from wt and the rest from MDV) [19] and 5∶3 (HA, NA and M or NS from *wt* and the rest from MDV) [17], [18] reassortants have been demonstrated to be prevalent and have to be eliminated from further cloning procedures. The clones containing the desired 6∶2 genes segments (in “pure” or mixed population) were subjected to the further cloning by limiting dilution and analyzed by pyrosequencing.

**Figure 3:**
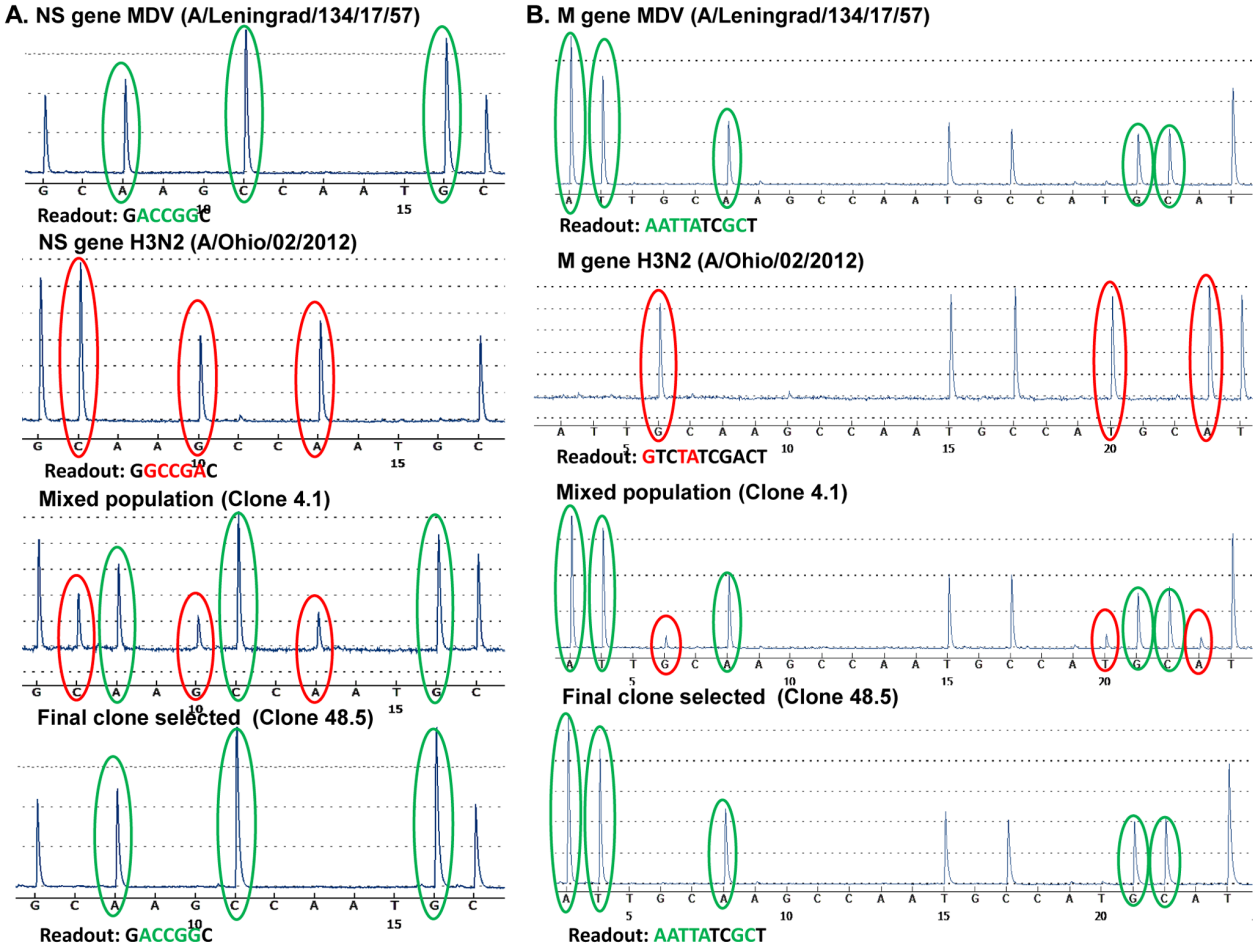
Pyrograms of NS (A) and M (B) genes of MDV (A/Leningrad/134/17/57 (H2N2), *wt* (A/Ohio/2/2012 (N3N2) and reassortant clones 4.1 and 48.5. The signature nucleotides of desired genes in the reassortants circled in green, non-desired – in red.

The viral reassortants obtained after selective passage of co-infection of MDV and A/Ohio/02/2012 (H3N2) virus were cloned using limiting dilution technique. Twenty nine clones were screened by pyrosequencing for NA, M and NS genes. The analysis showed that all reassortants had the NA gene of the desired *wt* origin. Only one clone was detected (number 4.1) which had the desired NS and M genes, but in a mixed population (Fig. 3A and 3B). Since only one candidate (clone 4.1) showed the presence of both NS and M genes of the desired MDV origin, RNA from this clone was subjected to further genotyping for the other four genes. The pyrosequencing analysis revealed that the rest of the internal genes of this clone had the correct MDV origin (Table 7). This clone was then subjected to the second round of cloning by a limiting dilution. Forty-six clones from the second cloning were analyzed by pyrosequencing for the NS and M genes. Forty-five of these clones had the M gene of the desired MDV origin, and only one clone had the *wt* M gene. All five clones from the highest limited dilution (10^−8^) contained the desired NS gene of MDV origin only, while the rest of the clones had this gene in a mixed population. One of these five clones with NS and M genes of correct MDV origin, clone 48.5 (Figure 3), was selected for further analysis of the remaining viral genes. Pyrosequencing analysis confirmed that the clone 48.5 contained the desired (6∶2) genome constellation (Table 7).

**Table 7:** Genome composition of reassortants between MDV and *wt*l viruses identified by pyrosequencing.

Virus used for reassortment	Clone number	Reassortment step			Gene	origin			
			HA	NA	NS	M	NP	PA	PB1
A/Victoria/361/2011 (H3N2)	6.4	1st cloning	wt	wt	MDV	MDV	MDV	MDV	MDV
A/Ohio/02/2012 (H3N2)	4.1	1st cloning	wt	wt	mix	mix	MDV	MDV	MDV
	48.5	2nd cloning	wt	wt	MDV	MDV	MDV	MDV	MDV
A/Texas/50/2012 (H3N2)	4.1	1st cloning	wt	wt	mix	mix	mix	mix	mix
	46.2	2nd cloning	wt	wt	mix	MDV	MDV	MDV	MDV
	467.6	3d cloning	wt	wt	mix	MDV	MDV	MDV	MDV
	4678.2	4th cloning	wt	wt	MDV	MDV	MDV	MDV	MDV
A/Anhui/1/2013 (H7N9)	2.3	selective	wt	mix	mix	mix	mix	MDV	MDV
	28.3	1st cloning	wt	wt	MDV	MDV	MDV	MDV	MDV

During the genotyping of reassortants between A/Victoria/361/2011 (H3N2) and MDV, the clone with a correct 6∶2 genome composition was detected at the stage of first cloning (clone 6.4). In contrast, it took four cloning procedures to find the reassortant with the correct 6∶2 genome composition in reassortment between MDV and A/Texas/50/2012 (H3N2) virus (Table 7). At the first cloning stage, all clones had a correct origin of NA gene but all other genes were present in a mixed population. Clone 4.1 was selected only because it had a higher proportion of NS-MDV gene present compared to other clones as was assessed by pyrosequencing.

**Figure 4:**
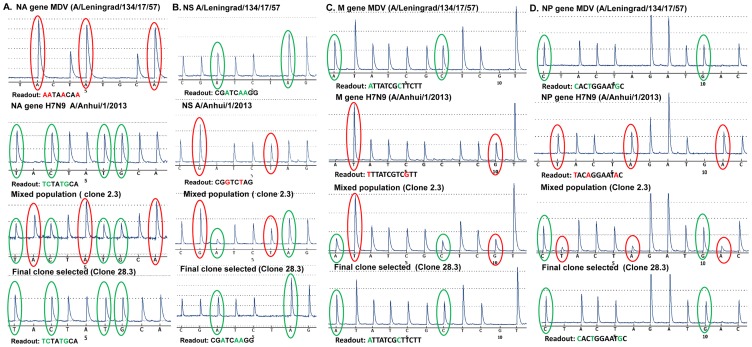
Pyrograms of NA (A), NS (B), M (C) and NP (D) genes of MDV (A/Leningrad/134/17/57 (H2N2) and *wt* (A/Anhui/1/2013 (H7N9) and reassortant clones 2.3 and 28.3. The signature nucleotides of desired genes in the reassortants circled in green, non-desired – in red.

The co-infection of MDV A/Leningrad/134/17/57 and *wt* A/Anhui/1/2013 (H7N9) virus was performed at different MDV:*wt* EID_50_ ratios: 10^−6^∶10^−7^, 10^−6^∶10^−8^ and 10^−6^∶10^−9^. The five eggs infected at selective conditions were then blindly passaged and genotyped for the origin of NA, NS and M genes. The pyrosequencing analysis showed that two eggs (2.3 and 2.4 both from MDV:wt ratio of infection 10^−6^∶10^−8^) had a reassortant virus pool with the desired gene combinations with NS and M from MDV virus and NA gene from *wt* (Figure 4). The pyrosequencing analysis of NP, PA, PB1 and PB2 for egg 2.3 and egg 2.4 showed that desired genes from MDV were prevalent (pyrogram for NP gene of egg 2.3 is shown in Fig. 4D). Progeny from egg 2.3 were cloned by limiting dilution in the presence of antiserum to MDV. Forty five clones derived from egg 2.3 were then analyzed for the origin of NA, NS and M gene. The pyrosequencing analysis showed that only two clones from the highest limited dilution 28.3 and 28.6 contained the desired pure *wt* NA and MDV NS and M genes. The pyrograms of NA, NS and M gene for *wt,* MDV, and pyrograms from one of these clones (28.3) are shown in Figures 4A, 4B and 4C, respectively. The pyrosequencing analysis of these clones identified that the rest of internal genes were of a desired MDV origin (Table 7).

We further cloned the selected 6∶2 reassortants for both influenza A (H3N2) and A (H7N9) two more times by limited dilution, and the presence of correct genome constellation in the clones was confirmed by pyrosequencing of all genes. These reassortant viruses were further subjected to genome stability analysis as described elsewhere [46], and the 6∶2 genome composition of vaccine candidate viruses was confirmed after five more passages in SPF eggs by real time RT-PCR analysis and complete genome sequence analysis. Both assays verified the origin of all RNA segments and the accuracy of pyrosequencing assay described here.

## Discussion

Every year, the WHO predicts the virus strains likely to circulate in the upcoming influenza season in each hemisphere and recommends vaccine virus strains to be included into the vaccine formulations. The timely delivery of vaccine doses before the coming influenza season is a high priority of vaccine manufacturers. The effectiveness is even more imperative in case of vaccine production for emergent viruses such as influenza A (H7N9) which poses a significant global health concern [47]–[49].

The generation of vaccine candidate virus strains, based on classical reassortment of wild-type and vaccine donor viruses, relies on the screening analysis for the identification of reassortant viruses with the desired 6∶2 genetic composition. The tight schedule associated with the production of the influenza vaccines necessitates an accurate and rapid method for genotyping reassortant viruses. There are a number of methods which have been used for reassortment detection, including real-time RT-PCR [50], conventional RT-PCR [25], [26], and conventional sequencing [51]. The most traditionally utilized methods for the genotyping of influenza reassortants for vaccine generation (RT-PCR-RFLP and multiplex RT-PCR) are accurate and reliable; however, both of these techniques have some disadvantages. For example, in the case of multiplex RT-PCR it is a difficult to design suitable primer combinations which enable amplification of all eight gene segments in a single-tube reaction [26]. In addition, in case of the high sequence homology between the genes of viruses used in reassortment, the genotyping by RT-PCR and RFLP at times could not be performed because of the lack of suitable restriction sites [29]. Pyrosequencing technique was shown to be an efficient approach for the subtyping of potential human influenza A virus reassortants. The report was based on the successful detection of the origin of all eight virus genes in laboratory virus isolates, original specimens and vaccine candidate virus reassortants [32].

In the present study we applied a pyrosequencing technique for the screening of LAIV reassortants generated in embryonated eggs. Since the desired 6∶2 genetic combination is often not predominant in the reassortant population during the cloning procedure, the large number of reassortants needs to be analyzed in order to select a correct genotype of the vaccine candidate [14], [18], [44]. To improve the vaccine candidate reassortant screening strategy, we used a two- step pyrosequencing approach which allowed the reduction of the number of analyzed reassortants at each step. The pyrosequencing technique allowed screening of 96 samples within a one hour run. This format allowed us to screen up to 30 reassortants for origin of NA, M and NS genes (first step), which are frequently involved in the generation of 7∶1 and 5∶3 reassortants [14], [16], [18], the selected clones (showing presence of correct NA, M and NS genes) were analyzed for the origin of the rest of the genes (second step).

Screening by the pyrosequencing assay has a number of advantages. First, it allows the detection of a mixed genotype populations providing sequence data which would discriminate even highly similar sequences. Second, the sensitivity of the pyrosequencing detected in the present study is high enough to detect as low as 5% of minor viral RNA in a mixture, this correlates well with the previous estimation of the minor variants in the mixed population detected by pyrosequencing [36], [40], [41]. This approach allowed us to exclude the allantoic clones with the abundant presence of genes of undesired origin in the mixed genotype at the first steps of the analysis and select the clones with the prevalent genes of desired origin for the following cloning procedure (Fig. 3A and 3B, clone 4.1; Fig. 4A and 4B, clone 2.3). In fact, our results of the reassortment analysis indicate that only a very few original clones contained the desired gene segments in a mixed population, and several sequential steps of cloning by limited dilutions are required for the selection of a reassortant with pure 6∶2 genome composition. Thus, our work indicates that pyrosequencing is an effective technique for the screening of virus genotypes and the selection of allantoic clones with a mixed virus population suitable for additional cloning.

In summary, our data demonstrated that the pyrosequencing assay is a sensitive, specific and reliable procedure suitable for rapid gene identification in the large number of reassortant clones created during LAIV candidate preparation. The higher sensitivity of the pyrosequencing method to detect mixed viral populations suggests that it could be used for quantitative assessment of the relative abundances of segment variants in a mixed population. The use of the pyrosequencing approach allowed rapid genotyping of the intermediate clones and generation of the LAIV candidates.
